# Approach for semi-automated measurement of fiber diameter in murine and canine skeletal muscle

**DOI:** 10.1371/journal.pone.0243163

**Published:** 2020-12-23

**Authors:** Courtney R. Stevens, Josh Berenson, Michael Sledziona, Timothy P. Moore, Lynn Dong, Jonathan Cheetham

**Affiliations:** Department of Clinical Sciences, Cornell University College of Veterinary Medicine, Ithaca, New York, United States of America; University of Minnesota Medical School, UNITED STATES

## Abstract

Currently available software tools for automated segmentation and analysis of muscle cross-section images often perform poorly in cases of weak or non-uniform staining conditions. To address these issues, our group has developed the MyoSAT (Myofiber Segmentation and Analysis Tool) image-processing pipeline. MyoSAT combines several unconventional approaches including advanced background leveling, Perona-Malik anisotropic diffusion filtering, and Steger’s line detection algorithm to aid in pre-processing and enhancement of the muscle image. Final segmentation is based upon marker-based watershed segmentation. Validation tests using collagen V labeled murine and canine muscle tissue demonstrate that MyoSAT can determine mean muscle fiber diameter with an average accuracy of ~92.4%. The software has been tested to work on full muscle cross-sections and works well even under non-optimal staining conditions. The MyoSAT software tool has been implemented as a macro for the freely available ImageJ software platform. This new segmentation tool allows scientists to efficiently analyze large muscle cross-sections for use in research studies and diagnostics.

## Introduction

Skeletal muscle is an adaptive tissue, which undergoes changes in mass and fiber composition in response to a wide range of stimuli including exercise, aging, trauma, as well as myopathic and neurological disease. Changes in muscle mass are primarily observed to be associated with atrophy or hypertrophy of individual myofibers as opposed to changes in fiber number [1,2]. Thus, characterization of fiber size distribution in the muscle tissue has significant diagnostic importance.

Muscle fiber size is routinely determined through imaging and analysis of fixed or frozen cross-sections. The fiber size distribution is typically quantified in term of cross-sectional area (CSA) or fiber diameter. The use of minimum Feret diameter is preferred as it is the least affected by distortion due to oblique cross-sectioning of muscle tissue [3].

The development of fluorescent immunohistochemistry (IHC) protocols which label the muscle fiber plasma membrane or extracellular matrix enable high contrast imaging of the fiber boundaries. Effective staining protocols for delineating muscle fibers include dystrophin [4], laminin [5], or collagen [6] staining techniques.

Despite availability of these labelling procedures to aid in identification of the fiber boundaries, segmentation and analysis of scans of muscle cross-sections is still most often accomplished using manual techniques. This is frequently done using basic image annotation software combined with a graphic tablet or mouse. This manual quantification process is tedious and time consuming [7]. Considerable regional variability in fiber size is often observed across a muscle section and so a large number of regions must be sampled across the specimen to accurately quantify the fiber size distribution in the overall muscle.

In attempts to speed up this process, several groups have described image-processing frameworks for the automatic segmentation and analysis of muscle cross-sections.

A typical image processing pipeline for muscle cross-sections requires several steps including pre-processing, segmentation, and morphological analysis [8]. The pre-processing step often involves re-adjusting intensity and contrast, background suppression, as well as to providing noise and artifact reduction of the original image. The segmentation step attempts to separate the muscle fibers from background. Finally, morphological analysis extracts feature data such as the fiber size distribution from the segmented image.

Common approaches for automated segmentation of muscle fiber cross-sections range from simple thresholding based strategies [7] to more advanced methods including active contour [9–13] and watershed based algorithms [6,14–17]. Unfortunately, while a number of image processing approaches to muscle cross-section analysis have been described in the literature, to date, only a limited number of research groups [13,14,16] have made their computer code available for general use by the research community.

In our experience, a common limitation of currently available software for automated analysis of muscle cross-sections is that segmentation accuracy tends to be highly sensitive to the quality of the input images. Technical issues include weak staining contrast, regional variations in intensity, non-specific staining, as well as presence of artifacts associated with freezing or sectioning [11]. These issues can result in mis-segmentation, which may require extensive manual correction. Because currently available software tools are so sensitive to these imaging conditions, their use has not yet gained broad acceptance as a practical tool for research studies and diagnostics.

In this paper, we present MyoSAT (Myofiber Segmentation and Analysis Tool), a semi-automated image processing pipeline that our group has developed to allow analysis of large (and even entire) muscle cross section images. Our goal in developing the MyoSAT software is to offer an improved method, which performs well even in cases of non-ideal staining conditions. The capability to analyze large regions of muscle enables the researcher to identify subtle changes in fiber size distribution between treatment groups.

## Materials and methods

### Tissue samples

Animal tissues used for development of the image analysis method were obtained as part of ongoing research studies associated with peripheral nerve repair. No animals were sacrificed specifically for purposes of this study.

#### Ethics statement

All associated research studies were performed in accordance with the PHS Policy on Humane Care and Use of Laboratory Animals, NIH Guide for Care and Use of Laboratory Animals, federal and state regulations, and was approved by the Cornell University Institutional Animal Care and Use Committee (IACUC, protocol #2012–0099).

#### Murine *tibialis-anterior* (TA) muscle tissue

Six C57 BL/6 mice (three male and three female) underwent a left side proximal-tibial to distal-common peroneal cross suture surgery with a conduit repair [18]. Five weeks after nerve injury, animals were euthanized and bi-lateral TA muscle cross sections were obtained.

#### Canine *crico-arytenoid lateralis* (CAL) muscle tissue

Five CAL muscle cross sections were obtained from each of two female beagle dogs with no history of upper airway disease and normal laryngeal function, which was determined endoscopically.

### Immunohistochemistry

The TA and CAL muscle sections were briefly fixed in cold acetone for 10 minutes. 8 μm cryosections were then obtained. The sections were washed in phosphate buffered saline containing 0.05% Tween 20 (PBST) and incubated with 10% rabbit serum and then goat anti-type V collagen antibody (Southern Biotech, Birmingham, AL) at 1:1000 for 1.5 hours. The sections were then further incubated with biotinylated rabbit anti-goat IgG (Vector Laboratories, Burlingame, CA) and streptavidin-Texas Red (Molecular Probe, Life Technologies, Grand Island, NY) to visualize staining. Finally, the sections were stained with DAPI and mounted in ProLong^®^ Diamond anti-fade mountant. As a negative control, goat IgG was diluted to the same final concentration as the primary antibody.

### Microscopy

The Murine TA cross sections were imaged using a Leica Aperio FL slide scanner at 20x. Image resolution: 0.462 μm/pixel (10bits/pixel). The images were saved in 16 bit TIFF format. Canine CAL cross sections were imaged using an Olympus AX 70 compound fluorescence microscope at 20x with an Optronics MicroFire camera. Image resolutions: 0.368 μm/pixel (12 bits/pixel). The images were saved in 16 bit TIFF format.

### Image processing development

The MyoSAT image-processing pipeline consists of 5 stages: 1) Intensity Leveling. 2) Contrast Enhancement. 3) Ridge Detection. 4) Ridge Image Post-Processing. 5) Watershed Segmentation.

#### Intensity leveling

**Fig 1:**
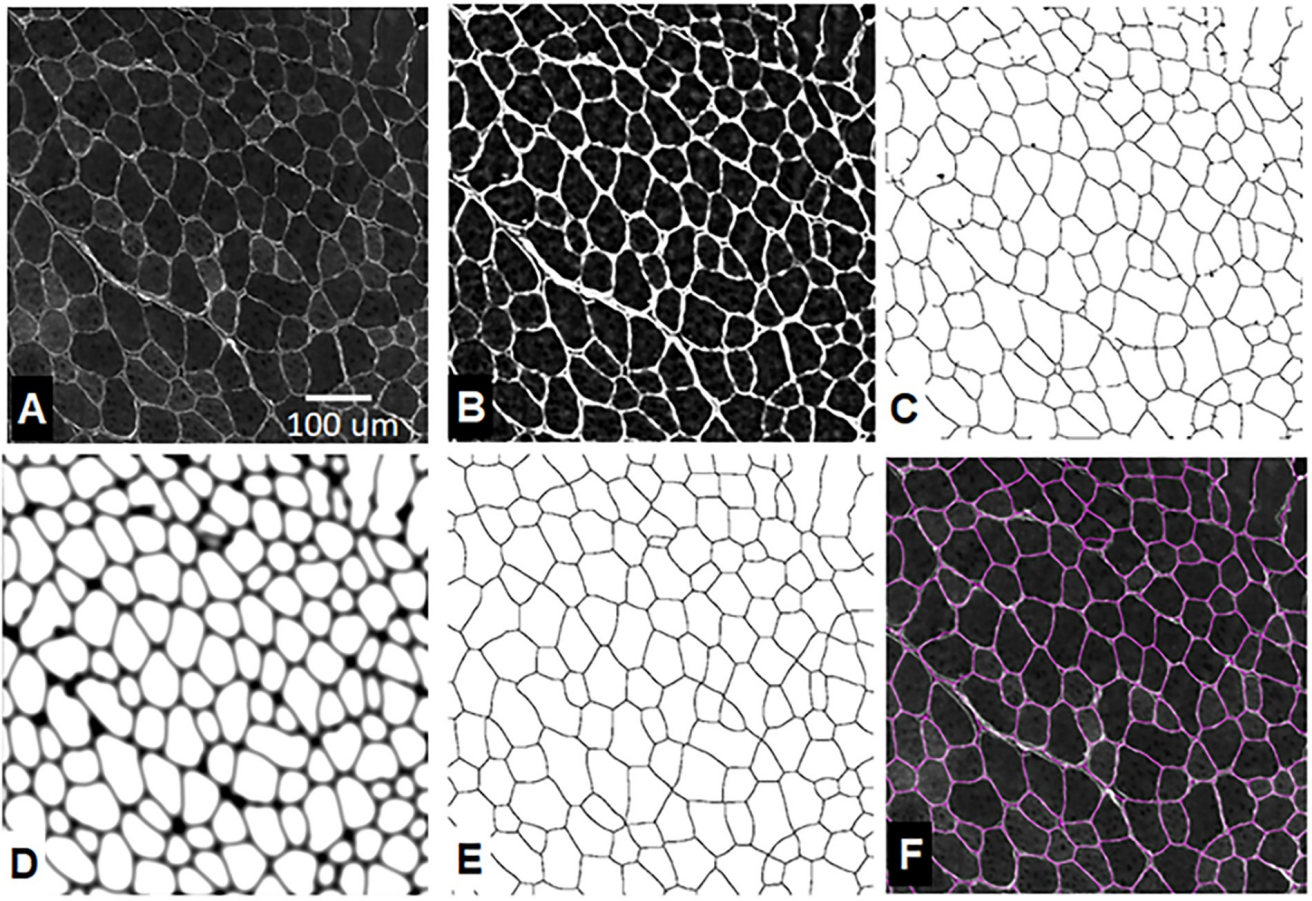
MyoSAT image processing steps: A) Original image. B) Intensity leveling and contrast enhancement. C) Ridge detection. D) Ridge image post-processing. [dilation + Gaussian blur + seed generation] E) Watershed segmentation. F) Result overlay.

In typical IHC stained muscle cross-sections, regional fluorescent intensity of the interiors of the muscle fibers is observed to vary across the sample due to non-specific staining as well as variations in tissue thickness. A background leveling technique is applied to suppress this variation. To accomplish this, first the background intensity of the fiber interiors is estimated by applying a median filter with kernel size of 45x45 μm. Any of the parameters discussed in this section can be adjusted in the macro. Custom values will be input in μm and calculated in pixels for use in the filters. This allows for use of images of different resolutions. Next, pixel values of the original image (Fig 1A) are divided by the median filtered image values, which results in a new “leveled” image with each pixel represented with a floating point value. Fiber interiors in this leveled image have a normalized average intensity of ~1.0 and the stained fiber boundaries have intensity >1.0.

#### Contrast enhancement

In the second stage, a Perona-Malik (PM) anisotropic diffusion filter [19] is applied to the new “leveled” image. This filter aids to suppress local image variations and pixel noise while preserving contrast of the fiber boundaries and to enhance the edges. After the PM filter step, the image pixel values are raised to the 4^th^ power which boosts the contrast of the extracellular membranes with respect to background (Fig 1B). The optimum value of the k parameter of the diffusion filter was experimentally found to obey a squared relationship, as shown below.

$$k=\;\frac{\sigma^{2}}{resolution{\left(\frac{\mu \text{m}}{\text{pixel}}\right)}^{2}}$$

Where σ is the filter width in μm. This was determined to be optimal at 3 for the validation images used. It can be adjusted to user preference.

#### Ridge detection

In the third stage, Steger’s line detection algorithm [20] is applied to locate the extracellular membranes between the muscle fibers. An appropriate scale factor (σ) is chosen to maximize detection of the fiber boundaries while rejecting image artifacts. The scale factor is related to the target boundary line width (*w*) by:

$$\sigma =\frac{w}{2\sqrt{3}}+0.5$$

Optimum value of σ was found to correspond to a target line width of 6μm. The macro calculates the value in pixels for use with images of different resolution. As with any parameter in the macro, this can be customized by the user. Output of the ridge detection algorithm is a binary “skeletonized” image containing the detected fiber boundaries (Fig 1C).

The thickness of the connective tissue is input into the macro as the sigma parameter of the ridge detection filter. The default value is the approximate average pixel width of the connective tissue in the validation images. It will be scaled linearly to the image’s resolution. This value can be adjusted for thicker or thinner connective tissue. Uneven thickness of connective tissue can cause incorrect segmentation over these areas. The most effective solution is to create a masking image that will prevent connective tissue areas from being included in the analysis.

#### Ridge image post-processing

The output image of the ridge detection algorithm often contains discontinuities as well as detected background artifacts. In order to convert the initial ridge detection image to a final segmented result, several additional post-processing steps are applied: First, a morphological dilation filter [21] using a disk shaped structuring element (radius = 6 μm) is applied to thicken the detected lines. The next step applies a gaussian blur filter to the binary image. Parameters for the morphological and Gaussian blur filters are also scaled linearly according to image resolution. In the final step, a manually adjusted threshold is applied to the blurred image such that values below the threshold are set to zero. The threshold is selected using a visual representation, showing all areas that will be considered below the threshold. The default value of 70 was experimentally selected and has been shown to produce accurate results with all images test. Adjusting the value can be used to fine tune results, helping correct for over- or under-segmentation. This step establishes seed locations for the watershed segmentation algorithm, which is described next (Fig 1D).

#### Watershed segmentation

The final stage of the pipeline applies a classic watershed segmentation algorithm [22] to the blurred image. Regions of zero intensity established by the manually adjusted thresholding step above provide seed locations for the segmentation algorithm. After watershed segmentation (Fig 1E and 1F), the detected objects are classified by cross-sectional area (CSA) and Feret diameter. To aid in filtering out mis-segmented regions, objects with CSA outside a predetermined range (200 to 10,000 μm^2^) are excluded from the analysis. This range corresponding to the smallest and largest fiber sizes observed in the target muscle tissues. Excluding values outside this range is used to reduce errors in MyoSAT. Through manual analysis, no fibers were measured outside this range. On inspection of results, segments larger than 10,000 μm^2^ typically correspond to groups of myofibers that were counted as a single object. Segments smaller than 200 μm^2^ correspond to artifacts that were mistakenly identified as small fibers. Within the macro, this range can be adjusted to user specifications. In the 22 validation images, on average approximately 3.3% of detected fibers below and 0.4% above this range. These fibers were not included in any analyses.

The image-processing pipeline has been implemented as a macro for the freely available image processing package FIJI / ImageJ [20]. The macro requires several third-party ImageJ plugins which implement several of the image processing algorithms [23–25]. Parameters for filters used in the Macro were selected and optimized using images at a 0.462 μm/pixel resolution. For images of different resolution, parameters for the median, Gaussian blur, line dilation, and ridge detection filters are defined in microns. These values are converted to pixels, which are used in the filters. The k parameter anisotropic diffusion filter follows a squared relationship with resolution.

### Validation

#### Validation image sets

The validation image sets consisted of 12 murine TA muscle cross-section images (ROI area: 0.17–0.86 mm^2^) and 10 canine CAL muscle cross-section images (ROI area: 0.26mm^2^).

#### Manual segmentation (Ground truth)

Fiber boundaries within the muscle cross-section images were manually segmented with the aid of a graphics tablet (Wacom Cintiq 22HD). Feret diameters of the manually segmented regions were then obtained using the ImageJ “Analyze Particles” function. The manual analysis was performed by one observer, not involved in the study.

#### SMASH software

To compare performance of the MyoSAT with other currently available open-source software for automated muscle cross-section image segmentation, the image sets were similarly segmented using the recently released SMASH (Semiautomatic Image Processing of Skeletal Muscle Histology) software package [16].

#### Accuracy analysis

Histograms of fiber diameter were generated for each muscle section image using each of the three segmentation methods (manual, MyoSAT, and SMASH). The accuracy of the automated approaches was compared to ground truth by statistical comparisons of mean fiber diameter obtained for each image. For reasons described in the previous section, segmented objects with CSA outside of the pre-determined given range (~200 to 10,000 μm^2^) were excluded from the analysis. Fibers intersecting the ROI edges were also excluded from analysis.

### Development of a staining contrast mapping tool

It was observed that several of the image-processing pipeline steps could be combined to provide a staining contrast mapping tool valuable for optimization of staining protocols. We define the staining contrast in the image as the intensity ratio between the staining of the fiber boundaries (extracellular membranes) to the non-specific staining of the fiber interiors.

The accuracy of most segmentation algorithms is often highly sensitive to staining contrast within the image. Such contrast information aids in quality control by identifying cross-section regions with sufficient contrast to provide good segmentation accuracy.

The three steps used to generate the staining contrast map are: 1) The original image is divided by the median filtered image to generate a “leveled image”. 2) The ridge detection image generated using Steger’s line detection algorithm is used as a mask to sample the centers of the fiber boundaries to generate the local contrast map. 3) An averaging function with kernel size (27 x 27 μm^2) is then applied to the map to reduce localized intensity variations. This aids in assessing overall staining contrast within a muscle region.

## Results

### Validation testing

The validation images were analyzed using three different segmentation methods: 1) Manual tracing (ground truth), 2) the proposed MyoSAT image processing method, and 3) the SMASH software package.

The image sets consisted of 12 murine TA muscle cross-section images containing 91–165 fibers (mean = 124.9); and 10 canine CAL muscle cross-section images containing 28–153 intact fibers per image (mean = 89.3)).

**Fig 2:**
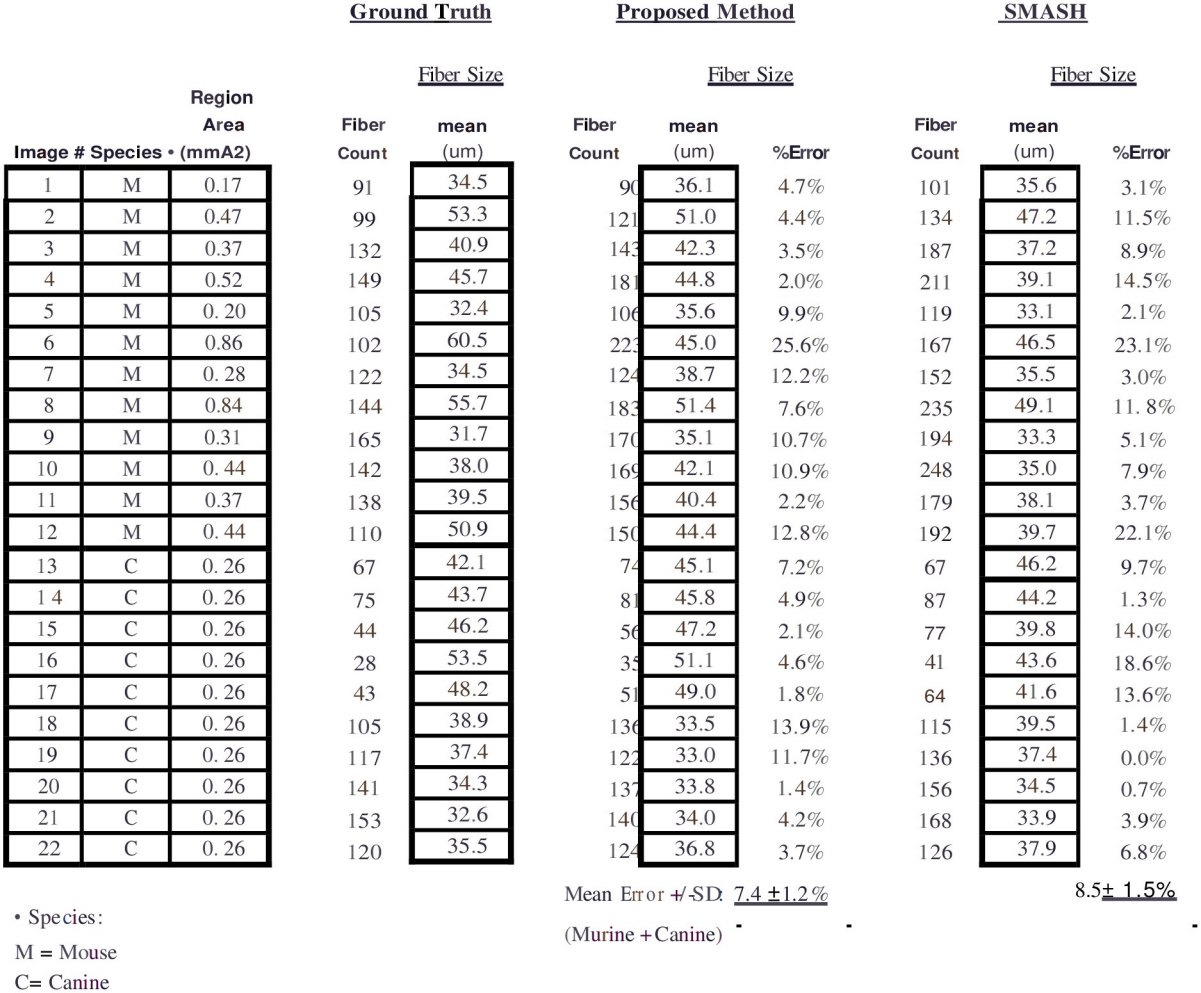
Comparison of manual tracing method (Ground truth), MyoSAT, and SMASH to analyze 12 murine (TA) and 10 canine (CAL) muscle cross-section images. (22 Images total) % Error = percent difference of the minimum Feret diameter from the ground truth.

We found that for both murine and canine tissue samples, fiber counts and fiber diameter obtained using the proposed semi-automated muscle segmentation method (MyoSAT) closely correlated to results obtained using manual segmentation of the muscle fibers (Fig 2).

As seen in Fig 2, some images have fiber counts greater than the ground truth. MyoSAT has a tendency to over-segment the image, rather than under-segment. The ridge detection filter, sometime recognizes artifacts as fiber divisions, causing single fibers to be subdivided and counted as multiple. Use of the noise reduction filters with appropriate parameters, described above, has reduced these errors. Artifacts are typically areas containing connective tissue or thicker sectioning, where fibers are less distinct. These errors have not been seen in clean areas with well-demarcated fibers. The quality of the muscle section has a strong effect on over-segmentation.

For the murine TA samples, no significant differences were observed between mean fiber estimates for the proposed method and ground truth (mean difference ± std error, 0.55 ± 0.94 μm, p = 0.57, paired two-tailed test). Significant differences were observed between ground truth and the SMASH method (3.03± 1.04μm, p = 0.0081, paired two-tailed test).

The average difference between the proposed method and ground truth for the 22 combined murine and canine sample images was 7.6% [SD = ±1.3%]. The difference between the SMASH method and ground truth was 8.5% [SD = ±1.5%]. We observed that the SMASH software had a tendency to over-segment the fibers resulting in higher fiber counts and underestimation of the fiber diameters.

**Fig 3:**
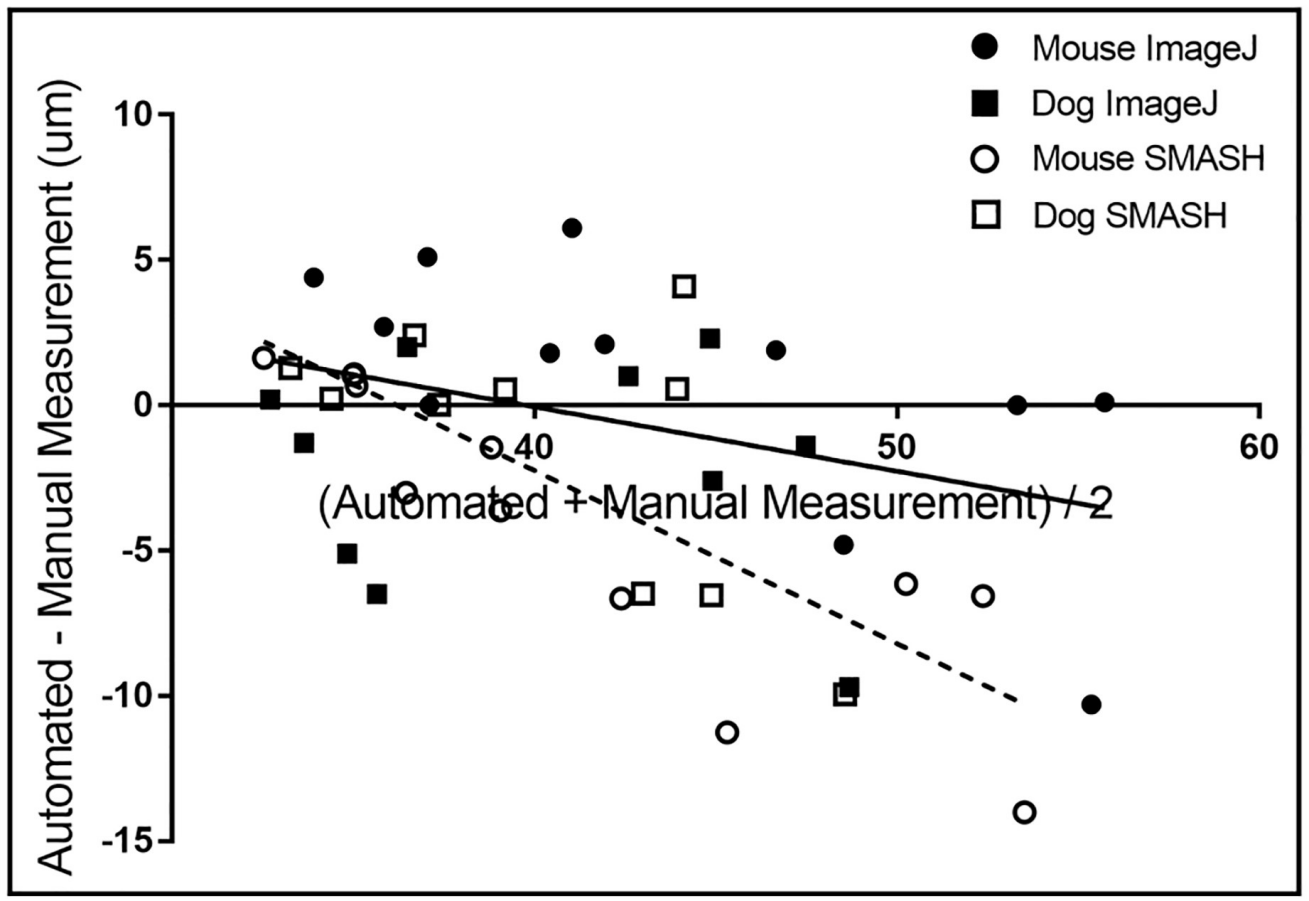
Combined Bland-Altman plot for murine and canine fiber diameter data obtained using MyoSAT, and SMASH compared to manual tracing method (Ground truth). Linear regression: MyoSAT: y = -0.22x + 8.8. SMASH: y = -0.58x +21.

Bland-Altman analysis [26] indicates that the MyoSAT method correlates slightly more closely (y = -0.22x + 8.8, p = 0.099) with the manual method than SMASH (y = -0.58x + 21, p = 0.0001, ANOVA, Fig 3).

We next compared the fiber size histograms generated using manual, MyoSAT, and SMASH segmentation methods. We observed that the MyoSAT analysis pipeline produces fiber size distributions histograms that more closely resemble ones generated by manual segmentation than size distributions generated using the SMASH segmentation approach.

### Analysis of full muscle cross-sections

A primary motivation for the development of the MyoSAT software was to develop a reliable method for segmentation and analysis of large muscle cross-sections regions.

**Fig 4:**
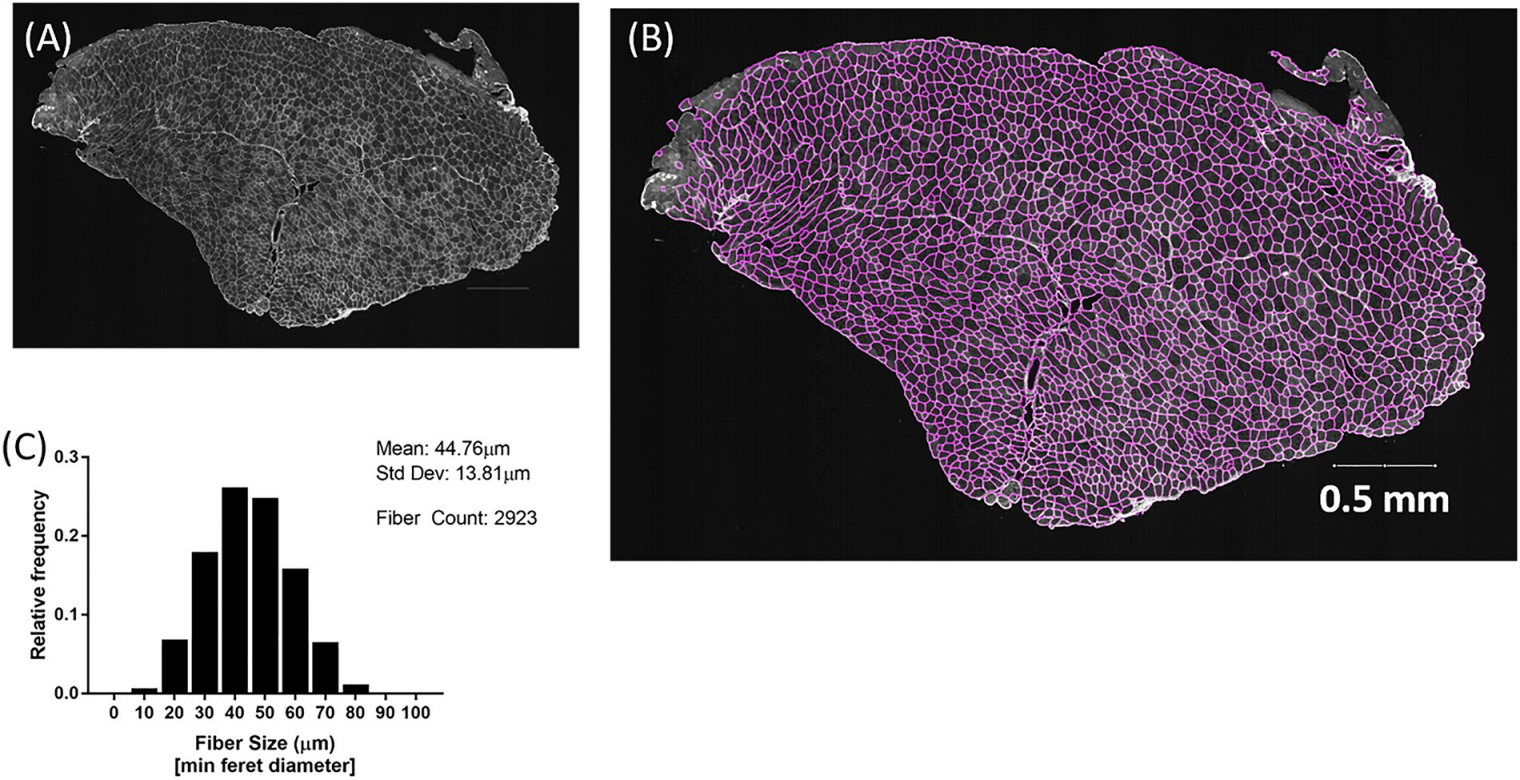
*A*,*B*:Example of full cross-section of murine TA muscle analyzed using the MyoSAT semi-automatic fiber detection software. *C*: Histogram of (min Feret) fiber diameters. MyoSAT has identified n = 2923 fibers with mean fiber diameter = 44.75μm (SD+/-13.81 μm).

Using the MyoSAT segmentation method to analyze entire murine TA muscle cross-section images frequently identified regions of non-uniform and low contrast staining. Fig 4 provides an example output of the proposed method. The analysis of the example image took approximately 15 minutes. We found analysis of such large regions to be unreliable using other available image processing software for automatic fiber segmentation due to issues including mis-segmentation, processing time, and image size constraints.

We next demonstrated the application of MyoSAT to identify changes in muscle physiology after injury. Left and right size TA muscle cross-sections were obtained from mice 5 weeks after a unilateral nerve transection. MyoSAT was used to analyze the full TA muscle cross-sections images containing between 1153–2637 identified fibers (mean 1784.1).

**Fig 5:**
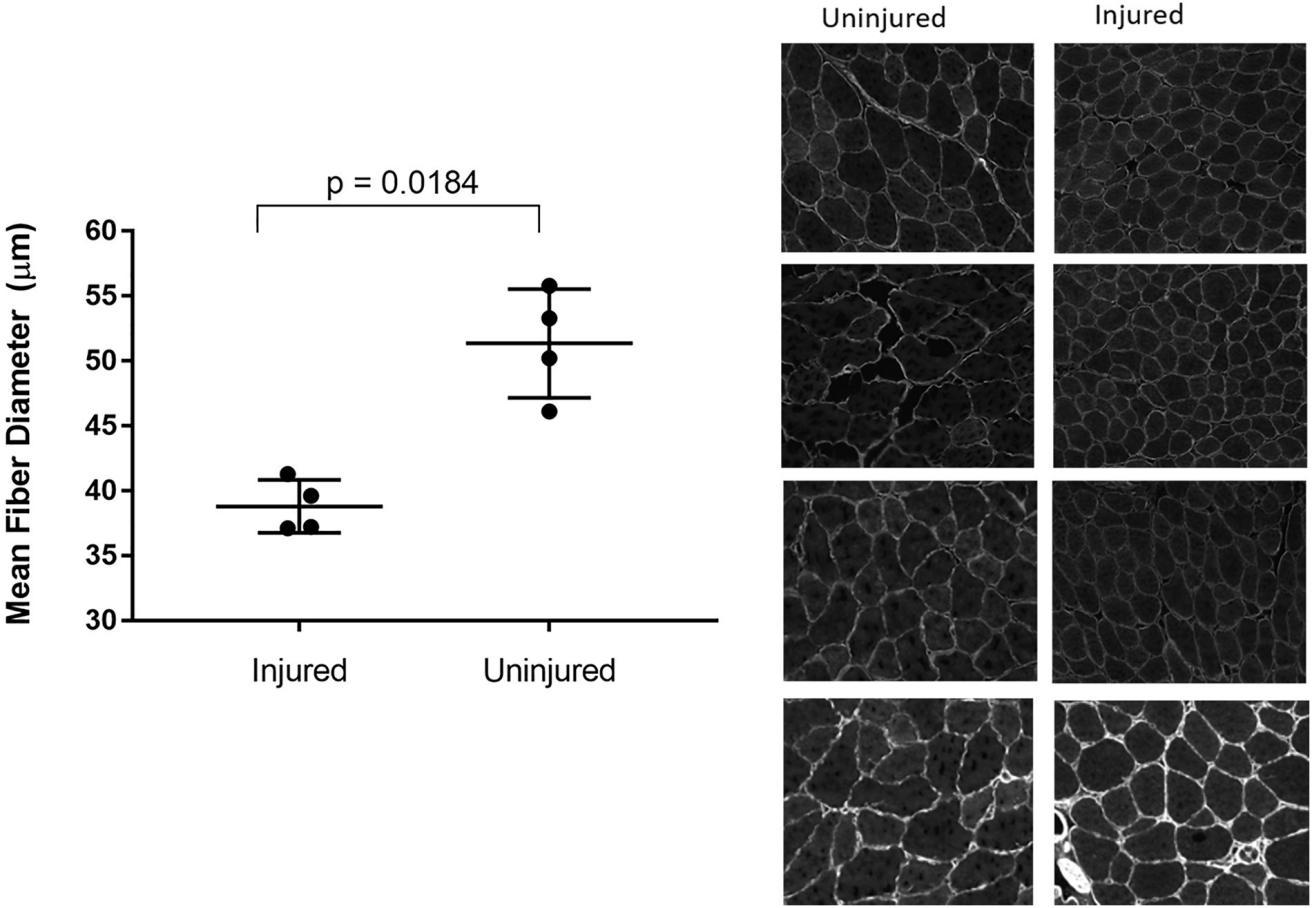
MyoSAT detects difference in muscle fiber diameter following nerve transection. Analysis used to evaluate change in muscle morphology in murine TA muscle 5 weeks after a common peroneal nerve transection. MyoSAT reveals significant differences in average fiber size between transected and uninjured sides. (n = 4 mice) Error bars = Std. Error.

As anticipated, evidence of reduced muscle fiber diameter was detected in the repaired limb (38.8μm (±1.0μm)) compared to the control (51.36 (± 2.1μm), Fig 5, p = 0.00184, n = 4 mice, paired t-test). This demonstrates the use of MyoSAT data to quantify and identify significant statistical differences in fiber size obtained via analysis of full-muscle cross-sections. This new method avoids the traditional approach of having to analyze a large number of individual ROI’s of each cross-section. The percentage of outliers, based on CSA (>10,000, <200 μm^2^) in the images were 3.5% below and 0.48% above, resembling the full validation image set.

### Application of contrast analysis tool

We found the staining contrast mapping tool, which we developed as a simple extension of the image-processing pipeline, to be very useful in providing an objective assessment of staining contrast. The contrast map allows the user to quickly identify poorly stained regions, which may result in reduced segmentation accuracy.

**Fig 6:**
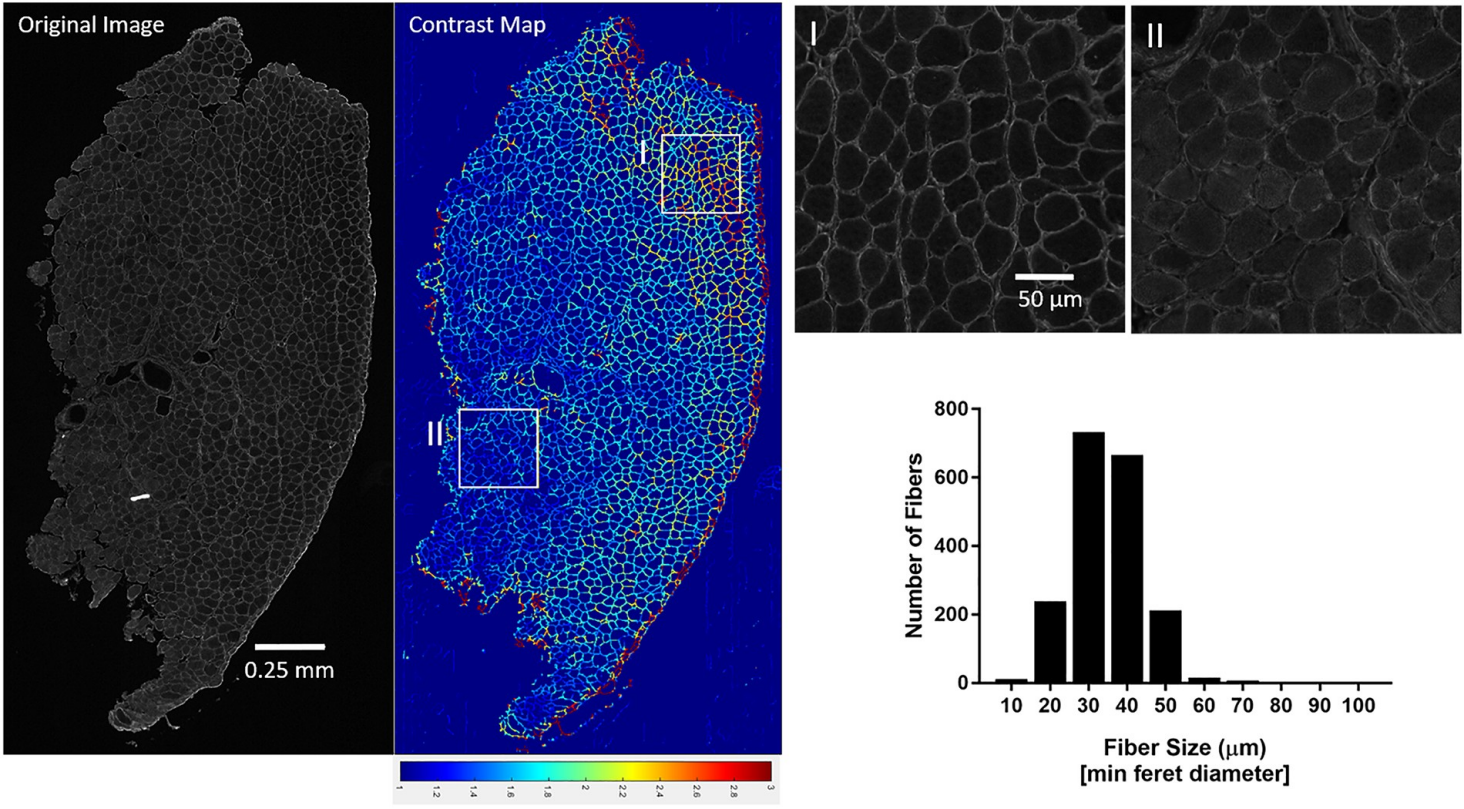
Development of contrast mapping tool to assess staining uniformity and contrast required for accurate fiber segmentation illustrating regions of high contrast (I) vs poor contrast (II) fiber staining. Contrast map range 1.0–3.0.

Fig 6 demonstrates output of the contrast-mapping tool applied to a full cross-section of Col V stained murine TA muscle exhibiting a non-uniform staining issue. In this case, the regional average contrast ratio between the fiber boundaries and the fiber interior ranged from approx. 1.0 to 3.0.

In our testing, a contrast ratio of approximately >2.25 across a region was found to provide consistent and accurate segmentation of the fiber boundaries using the MyoSAT software.

## Discussion

Here we have introduced a semi-automated image-processing pipeline (MyoSAT) which we developed for accurate segmentation and size distribution analysis of myofibers in muscle cross-section images. As we have described, MyoSAT incorporates several novel pre-processing steps to compensate for non-uniform staining intensity and to enhance the contrast of the fiber boundaries. Several of the unconventional steps within the image-processing pipeline include: 1) Aggressive pre-processing to compensate for non-uniform staining. 2) Application of anisotropic diffusion filtering to provide noise reduction and fiber border enhancement. 3) Use of Steger’s algorithm to detect and binarize the fiber boundaries.

Our validation tests have demonstrated that the MyoSAT analysis provides on average 92.4% accuracy for estimation of mean fiber diameter when compared to human segmented images in both murine and canine muscle tissue. Muscle fiber size distribution histograms generated during the analysis were found to closely approximate results obtained by manual segmentation.

It is important to note that the MyoSAT segmentation approach is based upon a line detection algorithm to detect the fiber boundaries, whereas most other software approaches are based upon edge detection algorithms. As a result, MyoSAT establishes segmentation boundaries halfway between the individual fibers instead of attempting to trace the fiber interior edges. As such, the proposed method has limited ability to reject the presence of excessive connective tissue in a region. Despite this limitation, the primary advantage of the line detection approach is the enhanced ability to segment low contrast images. This approach also avoids subjectivity associated with identifying the precise location of each fiber membrane edge, which is a common issue with the edge based detection algorithms.

MyoSAT is a semi-automated segmentation approach and so some manual adjustment of sensitivity parameters is required in order to achieve accurate segmentation. A disadvantage to this approach is that manual tuning can introduce some subjectivity into the analysis. However, an advantage is the ability to adjust the image processing to work for a wide range of imaging and staining conditions.

As with most automated image segmentation approaches, the segmentation accuracy of the proposed method is ultimately limited by staining contrast and image quality. A secondary outcome of our work has been the development of a contrast-mapping tool, which we have found to be useful for optimization and quality control of muscle staining protocols. The contrast mapping technique provides an objective approach to identify regions with sufficient staining contrast in order to yield accurate segmentation.

Our research group developed MyoSAT after having limited success with both commercial and open-source software packages to reliably analyze muscle fiber size distributions in large cross-sections, which we needed for own work. MyoSAT can be applied to entire cross sections of skeletal muscle, allowing the accurate segmentation and measurement of thousands of individual fibers.

Future development goals for the MyoSAT software include: 1) Continue to improve optimization of the image processing algorithms to improve performance. 2) Incorporate additional staining channel data to take advantage of ATPase [27] or myosin heavy chain [28] staining techniques to provide capability for fiber type distribution analysis. 3) Improve MyoSAT’s ability to handle images with areas of thicker connective tissue. 4) Test and improve MyoSAT’s accuracy under a variety of staining conditions.

MyoSAT has demonstrated it’s potential use for a wide range of range of studies including disease and regeneration models. The ability to rapidly measure muscle fiber diameter allows for fast assessment of nerve injury or muscle atrophy. While MyoSAT has only been tested using canine and murine muscles, it could be applied to skeletal muscle of any species. The availability of this software will enable the research community to efficiently analyze large muscle cross-sections for experimental studies and diagnostics.

## Supporting information

S1 Fig (TIF)

S2 Fig (TIF)
