# Supplementary figures and images for: Approach for semi-automated measurement of fiber diameter in murine and canine skeletal muscle
Source: PLoS One. 2020 Dec 23;15(12):e0243163. doi: 10.1371/journal.pone.0243163 (PMC7757813; doi:10.1371/journal.pone.0243163)

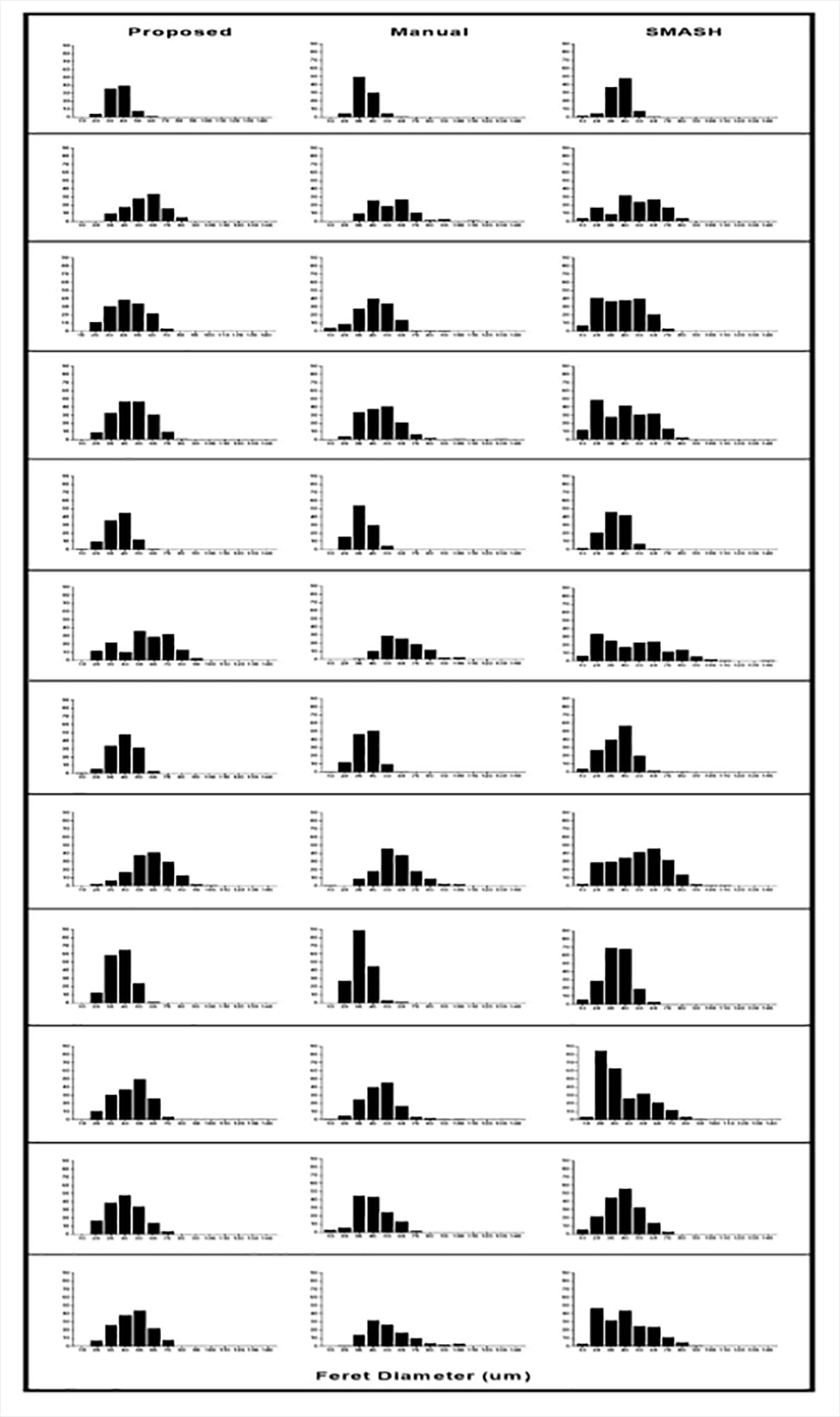

Supplement: S1 Fig — (TIF) [file pone.0243163.s001.tif]

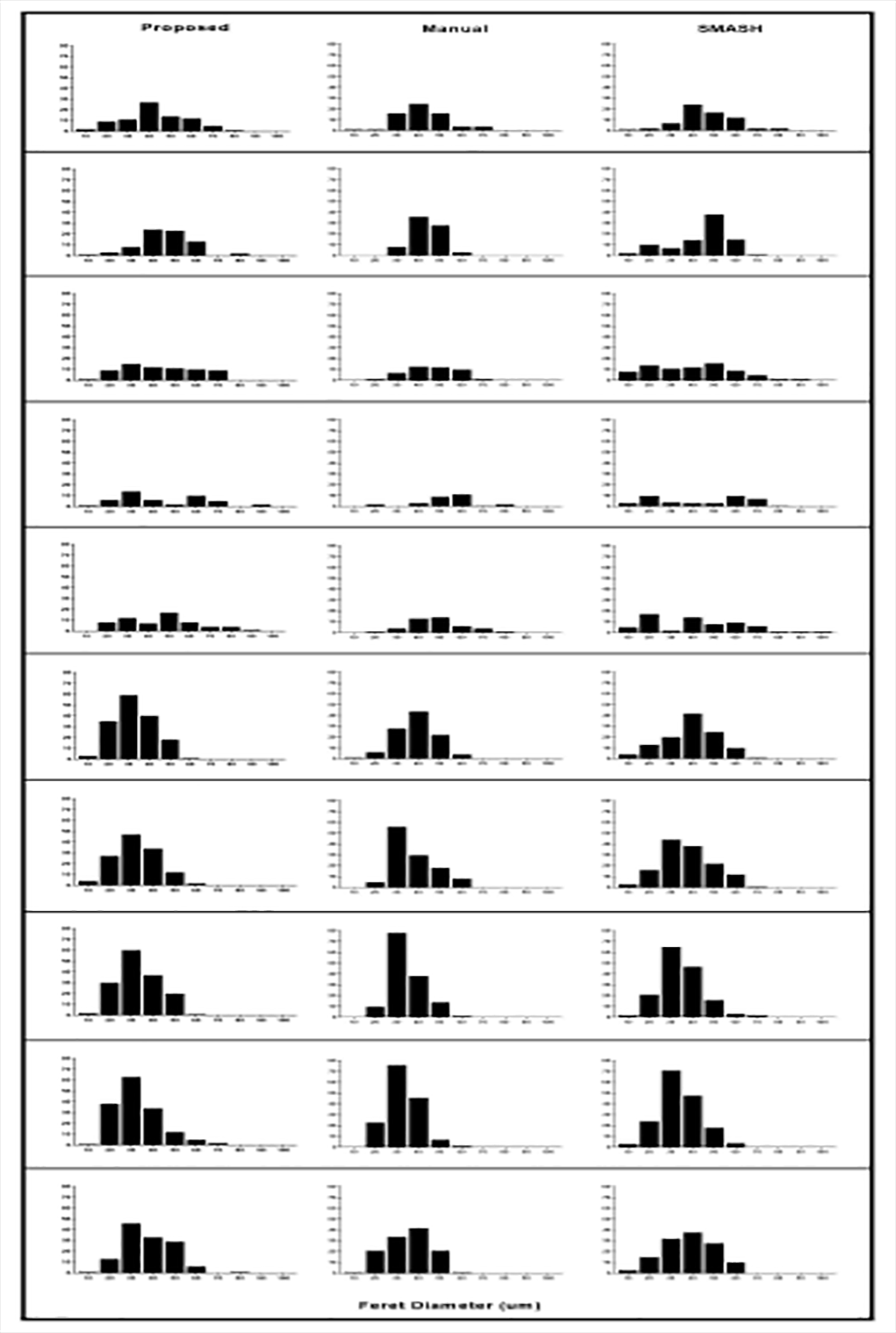

Supplement: S2 Fig — (TIF) [file pone.0243163.s002.tif]
